# A study of duration of digital dermatitis lesions after treatment in a Danish dairy herd

**DOI:** 10.1186/1751-0147-51-27

**Published:** 2009-07-01

**Authors:** Bodil H Nielsen, Peter T Thomsen, Jan T Sørensen

**Affiliations:** 1 Department of Animal Health and Bioscience, Faculty of Agricultural Sciences, Aarhus University, Blichers Allé 20, DK-8830 Tjele, Denmark

## Abstract

Digital dermatitis (DD) is a contagious disease of cattle affecting the skin adjacent to the claws. Disease dynamics of DD have been described to some extend, but we still need to quantify the duration of lesions and look into non-treatment factors affecting this. The aim of this study was to estimate the duration of lesions due to DD and to evaluate parity and lactation stage as potential risk factors for longer duration of such lesions. An estimate of the duration of lesions will be a valuable parameter in the evaluation of the economic impact of the disease and will additionally allow estimates of incidence based on prevalence figures. From May 2007 until November 2008, lesions associated with DD in the hind legs of 151 Danish Holstein cows at the Danish Cattle Research Centre were clinically scored on fifteen occasions. The mean interval between recordings was 39 days. Onset and end of each new case were estimated as midway between recordings prior to a change in the presence or absence of a lesion. Kaplan-Meier survival functions and Cox proportional hazard regression were performed to estimate the duration and analyse differences in the duration of lesions between primi- and multiparous cows and between different stages in lactation at onset of the lesion. The median duration of lesions were estimated to be 42 days, less than most previous published estimates. The relatively aggressive regime of topical treatment in the study herd might have shortened the duration of the lesions. Furthermore the comparatively long interval between recordings introduced an element of uncertainty in this estimate. No significant effects of parity or days in milk at lesion onset on the duration of DD were found using these data though lesions developed earlier in the lactation may have a longer duration. Further data would be needed to confirm the latter.

## Findings

Digital dermatitis (DD) is a contagious disease of cattle affecting the skin of the distal extremities. The inflammation causes varying degrees of irritation and pain and may cause severe lameness [1]. The transition between different stages of DD based on lesion development have been described [2-4] and in one study, the duration of a single case of DD has been reported to be approximately 70 days [5]. Somers *et al*. [4] reported that ulcerative lesions may persist for several months. An estimate of the duration of lesions would be a valuable parameter in the evaluation of the economic impact of the disease. Additionally, knowledge about the duration of lesions is of value in allowing estimates of incidences from prevalence data.

The aim of this study was to estimate the duration of lesions due to DD and to evaluate parity and lactation stage as risk factors for longer duration of such lesions. The hypotheses were that lesions in primiparous cows have a longer duration than in multiparous cows and that the duration of lesions occurring early in the lactation is longer than the duration of lesions with a later onset.

In the period from May 2007 until November 2008, DD associated lesions in hind legs of Danish Holstein (DH) cows (*N *= 151) at the Danish Cattle Research Centre, Faculty of Agricultural Sciences, Denmark, were clinically scored on 15 occasions with approximately five week intervals. The dairy herd was housed in a loose housing system with cubicles and slatted floor in the alleys as well as behind the feeding rack. At each recording all lactating and non-lactating cows were observed. Information about the individual animal (parity and days in milk (DIM)) was obtained from the herd database. Clinical examinations were performed by an experienced veterinarian or a trained technician. All examinations were done in a chute and the hind hooves were either fully trimmed or, where this was unnecessary, washed with water for recording. The lesions were recorded using a standardised scoring system [6] (see Table 1). If a lesion comprised of different stages of DD the leg was typed according to the most dominant stage. The leg was considered positive if any stage of DD (score 1,2,3,4 or 5) was present. All cases of DD score 1,2,3 and 4 were treated topically with a bandage containing salicylic acid (Salicylsyre, Jørgen Kruuse A/S, Langeskov, Denmark) that remained on the foot for 4 days and afterwards the lesion were topically treated on a daily basis with a spray containing chlortetracycline (Cyclospray, Novartis Health Care A/S, Copenhagen, Denmark) for 5 days. In order to estimate the duration of lesions only cases with a known onset and a known recovery were included.

**Table 1:** Scoring of lesions due to digital dermatitis according to reference [6]

Score	Description
0	No lesion
1	Hyperaemic area with erected pili
2	Moist, exudative and hyperaemic area with intact epidermis
3	Exudative area, exposed corium with no signs of healing
4	Exposed corium but in process of healing, dried-up lesion
5	Dark brown scab, completely or almost completely healed lesion

The onset of a case of DD was defined as a change from a negative recording (no lesions present) to a positive recording (DD like lesion present) from one recording to the next and the date of onset was set at the midpoint between these two recordings. Recovery of a case of DD was defined as the change from a positive recording to a negative recording from one recording to the next and the date of recovery as being equal to the midpoint between these two. The duration of lesions was calculated as the difference in days between onset and recovery. Cases that had an onset during the previous lactation were excluded. Only the first case seen in each cow was used. Data analyses were performed using the software SAS version 9.1 (SAS Inst., Inc., Cary, NC, USA). Kaplan-Meier survival functions (PROC LIFETEST) were performed initially to illustrate differences in the duration of lesions between primi- and multiparous cows and between three different stages in lactation at onset (stage 1: 0–120 DIM; stage 2: 121–240 DIM; stage 3: > 240 DIM). The effect of parity and DIM at onset of DD on the duration of the lesions was analysed using Cox proportional hazards regression (PROC PHREG). The time variable used in the model was days from onset of the first lesion until recovery. The predictors were parity group (primi- or multiparous) and DIM at onset of DD. The data contained tied event time and the EXACT method was used to approximate to the partial likelihood method.

During the study period, 1171 observations were obtained from 151 cows. The mean number of observations per cow was 7.8 (SD = 3.9). The mean recording interval was 39 days (SD = 13.1). The mean prevalence of lesions was 22.5% (SD = 8.1) and 20.0% (SD = 9.5) in the left and right hind leg, respectively. The censored data containing only the first DD cases with a known onset and recovery from each cow is described in Table 2.

**Table 2:** Description of censored dataset containing cases of digital dermatitis with a known onset and recovery date

N° of cases	
Total	68
	
Distribution of parity groups (%)	
Primiparous	75.0
Multiparous	25.0
	
Distribution of lactation stage groups (%)	
DIM* 0–120	35.3
DIM 121–240	38.2
DIM > 240	26.5

* days in milk

**Figure 1:**
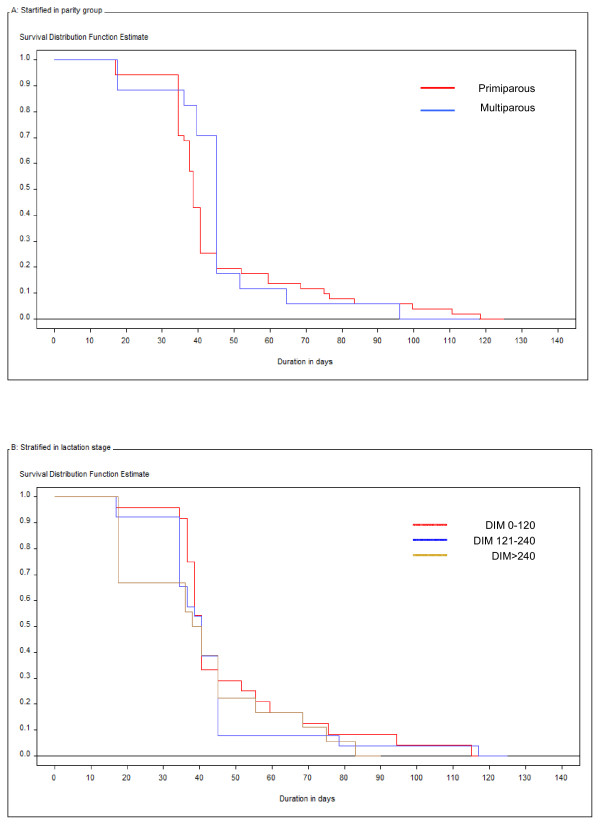
**Kaplan-Meier survival analysis plots for duration of lesions due to digital dermatitis**. A: Stratified by primi- and multiparous cows. B: Stratified by three lactation stages (0–120 days in milk (DIM); 121–240 DIM and > 240 DIM).

In Figure 1a &1b, Kaplan-Meier survival analysis plots for the duration of lesions are shown stratified by primiparous and multiparous cows and by three different lactation stages at time of onset, respectively. The median duration of lesions was 42 days (Table 3). No effects of parity group or lactation stage were found on the median durations. The hazard ratios found in the Cox regression analysis were 0.698 (*P *= 0.21) and 1.002 (*P *= 0.14) for parity group and DIM at lesion onset, respectively.

**Table 3:** Median duration of digital dermatitis associated lesions in days. Interquartile range shown in brackets

	Median duration
No stratification	42 (36–48)
	
Stratification	
Primiparous	39 (35–48)
Multiparous	48 (42–48)
	
Lactation stage at lesion onset	
DIM* 0–120	42 (39–56)
DIM 121–240	42 (35–48)
DIM > 240	41 (35–48)

* days in milk

The median duration of DD associated lesions found in the present study is shorter than the 70 days found in one case [5] and the persistence of ulcerative lesions previously observed [4]. The interval length between recordings (mean 39 days) might have resulted in some inaccuracy in this estimate. Moreover, the regime of topical treatment of lesions in this particular herd is considered relatively aggressive and may have resulted in a shorter duration than expected in commercial herds in general. If there was indeed an effect of treatment, the duration of lesions estimated in the present study would clearly be an underestimate of the duration of a non-treated case.

Primiparous cows have been found to have a greater likelihood of incomplete healing of DD associated lesions and it was suggested that duration of lesions might be prolonged in primiparous compared to multiparous cows [7]. In the present study, we found no effect of parity on the duration of lesions. However, in this herd heifers were housed with lactating cows and therefore did not experience the considerable change in environment that often occurs for this group of animals around calving with their introduction to the main milking herd.

The increased risk for infectious diseases in early lactation has been found true for DD in some studies [8,9], whereas others as here not have been able to recognise DIM as an risk factor for DD [10,11]. It might be speculated that lesions occurring late in the lactation have a shorter duration than lesions occurring in early lactation where the metabolic stress culminates and there is a trend for this here. This area needs further study

In conclusion, the median duration of lesions due to DD which were treated aggressively was estimated as 42 days and our first hypothesis of an increase in the duration of lesions in primiparous compared to multiparous cows was not proven. However, there was a trend towards an increased duration of lesions that occurred in early lactation and we suggest that this hypothesis is not dismissed without further study.

## Competing interests

The authors declare that they have no competing interests.

## Authors' contributions

BHN carried out some of the clinical examinations, participated in the design of the study, performed the statistical analysis and drafted the manuscript. PTT and JTS participated in the design of the study and helped to draft the manuscript. PTT also participated in the clinical examinations. All authors have read and approved the final manuscript.
